# The value of decreasing the duration of the infectious period of severe acute respiratory syndrome coronavirus 2 (SARS-CoV-2) infection

**DOI:** 10.1371/journal.pcbi.1008470

**Published:** 2021-01-07

**Authors:** Bruce Y. Lee, Sarah M. Bartsch, Marie C. Ferguson, Patrick T. Wedlock, Kelly J. O’Shea, Sheryl S. Siegmund, Sarah N. Cox, James A. McKinnell

**Affiliations:** 1 Public Health Informatics, Computational, and Operations Research (PHICOR), City University of New York Graduate School of Public Health and Health Policy, New York City, New York, United States of America; 2 Infectious Disease Clinical Outcomes Research Unit (ID-CORE), Lundquist Institute, Harbor-UCLA Medical Center, Torrance, California, United States of America; 3 Torrance Memorial Medical Center, Torrance, California, United States of America; Institute for Disease Modeling, UNITED STATES

## Abstract

Finding medications or vaccines that may decrease the infectious period of severe acute respiratory syndrome coronavirus 2 (SARS-CoV-2) could potentially reduce transmission in the broader population. We developed a computational model of the U.S. simulating the spread of SARS-CoV-2 and the potential clinical and economic impact of reducing the infectious period duration. Simulation experiments found that reducing the average infectious period duration could avert a median of 442,852 [treating 25% of symptomatic cases, reducing by 0.5 days, reproductive number (R_0_) 3.5, and starting treatment when 15% of the population has been exposed] to 44.4 million SARS-CoV-2 cases (treating 75% of all infected cases, reducing by 3.5 days, R_0_ 2.0). With R_0_ 2.5, reducing the average infectious period duration by 0.5 days for 25% of symptomatic cases averted 1.4 million cases and 99,398 hospitalizations; increasing to 75% of symptomatic cases averted 2.8 million cases. At $500/person, treating 25% of symptomatic cases saved $209.5 billion (societal perspective). Further reducing the average infectious period duration by 3.5 days averted 7.4 million cases (treating 25% of symptomatic cases). Expanding treatment to 75% of all infected cases, including asymptomatic infections (R_0_ 2.5), averted 35.9 million cases and 4 million hospitalizations, saving $48.8 billion (societal perspective and starting treatment after 5% of the population has been exposed). Our study quantifies the potential effects of reducing the SARS-CoV-2 infectious period duration.

## Author summary

Finding medications or vaccines that may decrease the infectious period of severe acute respiratory syndrome coronavirus 2 (SARS-CoV-2) could potentially reduce transmission in the broader population. We developed a computational model of the U.S. simulating the spread of SARS-CoV-2 and the potential clinical and economic impact of reducing the infectious period duration. Our simulation experiments found that reducing the average infectious period duration could avert a median of 442,852 to 44.4 million SARS-CoV-2 cases, varying the proportion of cases treated, average duration of the infectious period, and the reproductive rate. At $500/person, treating 25% of symptomatic cases saved $209.5 billion (societal perspective, R_0_ 2.5). Further reducing the average infectious period duration by 3.5 days averted 7.4 million cases (treating 25% of symptomatic cases). Expanding treatment to 75% of all infected cases, including asymptomatic infections (R_0_ 2.5), averted 35.9 million cases and 4 million hospitalizations, saving $48.8 billion (societal perspective and starting treatment after 5% of the population has been exposed). Our study suggests that finding ways to reduce the infectious period of SARS-CoV-2 could help decrease its spread and impact.

## Introduction

Finding medications or vaccines that may decrease the infectious period of severe acute respiratory syndrome coronavirus 2 (SARS-CoV-2) could benefit not only those who receive the medications or vaccines but potentially reduce transmission in the broader population. For example, studies have suggested that Remdesivir may reduce the time to recovery in patients with severe COVID-19 infections.[1, 2] A question then is what might be the broader population effects and impact of reducing the length of time that a person may shed the virus.[3] Therefore, we developed a computational model simulating the spread of SARS-CoV-2 and the potential clinical and economic impact of reducing the average infectious period duration.

## Results

### Decreasing the average infectious period by 0.5 days

#### Only targeting symptomatic cases

**Fig 1:**
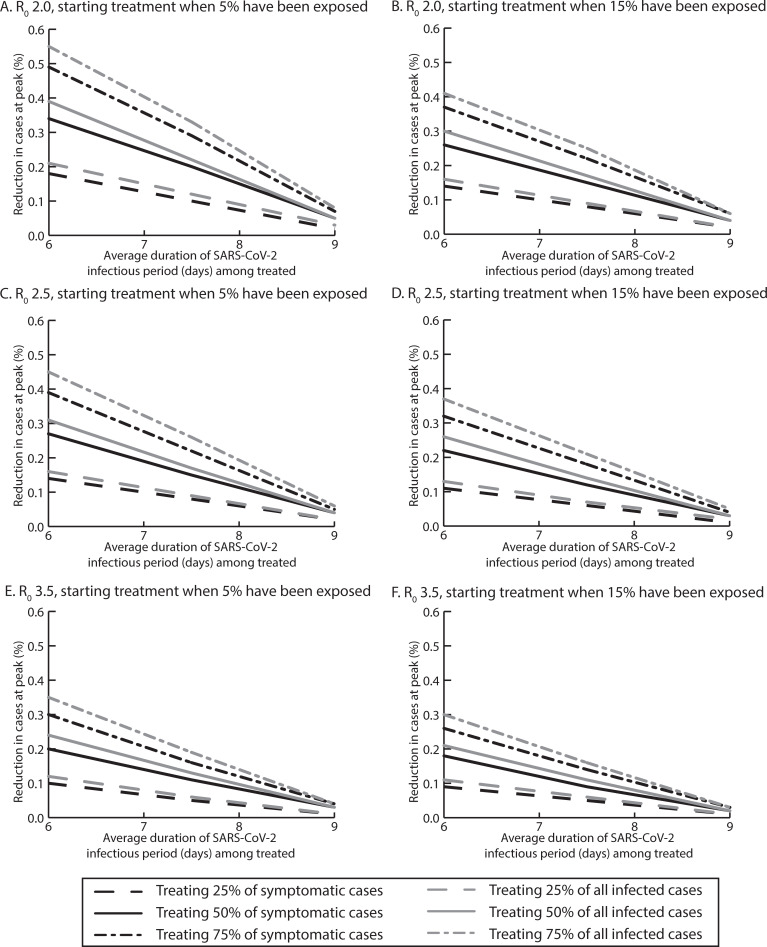
Percent reduction in SARS-COV-2 cases at the epidemic peak for various reductions in the average infectious period duration when treating various proportions of symptomatic and all infected cases with a reproductive rate (R_0_) of A) 2.0, when starting treatment after 5% of the population has been exposed, B) 2.0, when starting treatment after 15% of the population has been exposed, C) 2.5, when starting treatment after 5% of the population has been exposed, D) 2.5, when starting treatment after 15% of the population has been exposed, E) 3.5, when starting treatment after 5% of the population has been exposed, and F) 3.5, when starting treatment after 15% of the population has been exposed.

**Fig 2:**
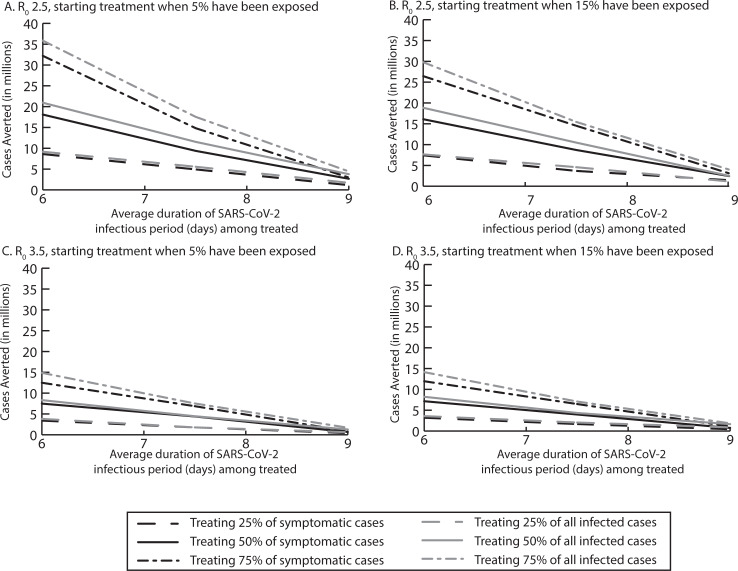
Median number of SARS-CoV-2 cases averted by reducing the average infectious period duration when treating various proportions of symptomatic and all infected cases when A) starting treatment after 5% of the population has been exposed with an R_0_ of 2.5, B) starting treatment after 15% of the population has been exposed with an R_0_ of 2.5, C) starting treatment after 5% of the population has been exposed with an R_0_ of 3.5, and D) starting treatment after 15% of the population has been exposed with an R_0_ of 3.5.

**Fig 3:**
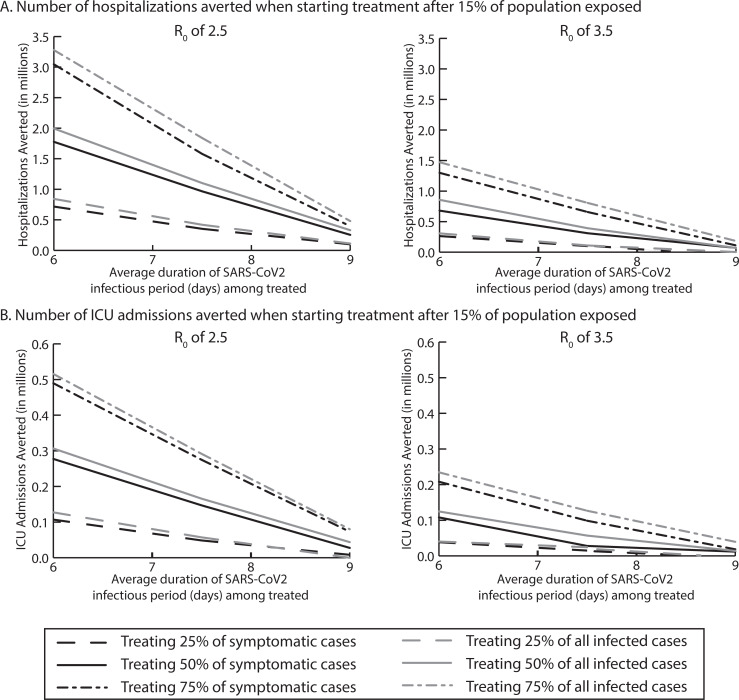
Median number of A) hospitalizations averted, and B) ICU admissions averted, by reducing the average infectious period duration when treating various proportions of symptomatic and all infected cases when starting treatment after 15% of the population has been exposed with an R_0_ of 2.5 and 3.5.

Treating all symptomatic COVID-19 cases (average infectious period reduced by 0.5 days to an average duration of 9 days) with an reproductive number (R_0_) of 2.5, was enough to reduce the peak of the epidemic curve (Fig 1), averting 1.4–3.1 million cases (Fig 2). Fig 3 shows the impact on healthcare use, which further averted 916,218 total bed days (of which 302,085 were ventilated bed days), 254,118 hospitalizations and 27,623 ICU admissions when treating 50% symptomatic cases (Fig 3B).

When the entire treatment course cost $500, it was not cost-effective or cost saving to third party payers, however it was cost saving from the societal perspective (e.g., $209.5 billion treating 25% of cases, R_0_ 2.5). When medication/vaccination cost $2,500 there were still net cost savings from the societal perspective when 50% of symptomatic cases were treated ($44.9 billion).

Varying R_0_ (3.5–2.0) averted anywhere from 0.44–4.6 million cases. For example, at R_0_ 3.5, treating 25% to 75% of symptomatic cases reduced the epidemic curve peak and averted 0.4–1.1 million cases. Treating 75% of patients averted 1 million total bed days (of which 32,531 were ventilated bed days), 111,418 hospitalizations, and 18,524 ICU admissions (Figs 1, 2 and 3). When the treatment cost $500 for the entire course, treatment was cost-effective from the third-party perspective, [for example, when 75% of symptomatic cases were treated ($3,388/QALY saved)] and was cost saving from a societal perspective ($113.5 billion). At R_0_ 2.0, treating 25% to 75% of symptomatic patients averted 1.5 million-4.6 million cases and 37,922–536,553 hospitalizations, and was cost-effective when 25% or 50% were treated ($53- $17,723/QALY saved) from the third party payer perspective and was cost-saving from the third-party payer ($3.8 billion) and societal perspective ($76.2 billion) when 75% were treated ($500 per person cost).

#### Targeting all infected with SARS-CoV-2

With R_0_ 2.5, treating all infected cases averted 56,872 more SARS-CoV-2 cases than treating all symptomatic cases (treating 50% Fig 2B). However, even treating only 25% of those infected averted 1.1 million cases and 112,045 hospitalizations. A $500 total treatment cost per person was cost saving ($498.8 million) when treating 50% of all infected from the third-party payer perspective. When treating 75% of all infected, treatment saved ≥$327 billion from the societal perspective.

With R_0_ 3.5, treating 25%-75% of all cases averted 432,978–750,059 additional cases and was cost-effective when at least 50% of all cases were treated (≤$7,891/QALY saved) from the third-party payer perspective. With R_0_ 2.0, treating 25%-75% of all infected averted 2.3–7.0 million cases and 165,835–778,241 million hospitalizations. Treating 75% of all infected saved $9.6 billion from a third-party payer perspective and $458.9 billion from a societal perspective (treatment cost $500 per person).

### Decreasing the average infectious period by 2 days

#### Only targeting symptomatic cases

Decreasing average infectious period duration by 2 days to an average infectious period duration of 7.5 days, averted 3.7–14.5 million cases (Fig 2). For example, treating 50% of symptomatic cases averted 8.7 million SARS-CoV-2 cases, 966.9 million hospitalizations, 147,066 ICU admissions (Fig 3), and 756,494 ventilated bed days. At a total cost of $500 per person, reducing the infectious period duration saved ≥$8.8 billion and ≥$172 billion from the third-party payer and societal perspectives, respectively.

At R_0_ 3.5, treating 25%-75% of symptomatic cases averted 1.6–6.5 million SARS-CoV-2 cases and 131,469–657,423 million hospitalizations, saving third-party payers $2 billion and society $544.2 billion when 75% are treated ($500/person treatment cost). At R_0_ 2.0, treating 25%-75% of symptomatic cases averted 7.0–20.6 million cases and 620,092–2.3 million hospitalizations, saving $6.4-$32.1 billion and $170.9-$719.1 billion from the third-party payer and societal perspectives, respectively.

#### Targeting all infected with SARS-CoV-2

Expanding treatment to asymptomatic cases averted 4.6–13.9 million cases, varying with the proportion treated (R_0_ 2.5). For example, treating 50% of those infected averted 10.5 million cases, 1.1 million hospitalizations, and 165,783 ICU admissions, saving ≥$9.3 billion and ≥$194.1 billion from the third-party payer and societal perspectives, respectively, when the total treatment course was $500 per person.

When R_0_ is 3.5, treating 25–75% of all infections averted 2.0–7.1 million SARS-CoV-2 cases and 110,012–806,252 hospitalizations, and when treating 50% of cases it is cost-effective ($654/QALY saved) for third-party payers and saved $219.4 billion for society with a $500 total treatment cost per person. With R_0_ 2.0, treating 25–75% of all infections averted 6.5–24.6 million SARS-CoV-2 cases and was cost saving from both the third-party and societal perspectives with a total treatment cost of $500 per person (saving $194.7-$626.9 billion from the societal perspective).

### Decreasing the average infectious period by 3.5 days

#### Only targeting symptomatic cases

Further reducing the average infectious period by 3.5 days to an average duration of 6 days, averted 7.4–26.4 million cases when treating 25% to 75% of symptomatic cases (R_0_ 2.5; Fig 2). Treating 75% of cases averted 3 million hospitalizations (Fig 3) and 370,945 patients from requiring a ventilator, saving ≥$36.4 billion and ≥$757.3 billion from the third-party payer and societal perspectives, respectively, when treatment cost $500 per person.

As Fig 2 shows, varying R_0_ (2.0–3.5) and the proportion of symptomatic cases treated averted 0.44–40.5 million SARS-CoV-2 cases. For example, with R_0_ 2.0, treating 25% of symptomatic cases averted 11.1 million cases, and treating 50% of symptomatic cases averted 26.4 million cases, saving $600.6 billion from the societal perspective ($37.6 billion from the third-party payer perspective). At R_0_ 3.5, treating 50% of symptomatic cases averted 0.81 million cases, and was cost-effective from the third-party payer perspective ($3,715/QALY saved) and cost savings from the societal perspective ($326.7 billion) when it cost $500 for the full treatment course.

#### Targeting all infected with SARS-CoV-2

Treating all infected cases substantially reduced the peak of the epidemic curve (Fig 1). This averted 7.7–29.7 million cases, depending on proportion treated (R_0_ 2.5). For example, treating 75% of all infections averted 3.3 million hospitalizations, saving $856 billion (societal perspective) when the entire treatment course cost $500 per person. At R_0_ 3.5, treating 75% of all infected averted 14.1 million cases, saving $11 billion and $427.2 billion from the third-party payer and societal perspectives, respectively. With R_0_ 2.0, treating 25% of the population averted 12.7 million cases, and treating 75% of the population averted 44.4 million cases, saving ≥$67.3 billion and ≥$1.2 trillion with a ≤$500 total treatment cost per person from the third-party payer and societal perspectives, respectively.

## Discussion

Our study suggests that finding ways to reduce SARS-CoV-2’s infectious period could help decrease its spread and impact. Theoretically, decreasing the period during which a person is infectious can decrease the number of effective exposed contacts (i.e., contacts in which transmission of the virus occurs) over the course of their infection. Findings from our study show when medication or vaccination reduces the duration of the infectious period by at least 0.5 days, even treating 25% of symptomatic cases leads to a reduction in cases and cost savings from the societal perspective, but does not substantially affect hospital resource use (e.g., ICU admissions, ventilated bed days) and is not cost-effective from the third party payer perspective ($500 per person treatment cost). However, treating 75% of symptomatic cases is cost-effective or cost savings from both the third-party payer and societal perspectives. When a treatment reduces the infectious period by 2 days or more, providing the treatment to even 25% of symptomatic cases reduces hospital resource use and generates cost savings from the third-party payer and societal perspectives when treatment costs $500. Assuming that the virus’ inherent contagiousness and the number of daily social contacts remains the same, less time to transmit the virus would mean fewer new cases generated. This also could affect the herd immunity threshold: the proportion of a population that needs to be immune to the virus so that it can no longer spread. The herd immunity threshold is directly related to the number of new cases generated by each infected person, as seen by the following equation: 1-(1/R_0_).[4, 5] As the pandemic progresses and more people recover from infection and become immune, it becomes more difficult for infectious individuals to encounter susceptible people. Similarly, decreasing the infectious period gives people who are infectious even less time to encounter and transmit the virus to those susceptible. This window to encounter susceptible people decreases more and more as the infectious period duration is further reduced.

The relationship between the infectious period duration and the herd immunity threshold does depend on how many people for which this duration is reduced. Our results show that reducing the infectious period for 25% of those who are symptomatic can avert 0.44–11.1 million cases, while expanding to all infected cases averted an additional 0.46–1.6 million cases. Assuming that asymptomatic cases transmit less than symptomatic cases, our estimates for the benefits of treatment are more conservative. The amount of cost savings estimated is the ceiling amount that could be invested into expanding access to treatment (e.g., through contact tracing, subsidization) and still break even with the investment. Ultimately, the percentage of infected patients that can be treated will depend on the availability and implementation of testing, access to healthcare, and the production and delivery of medications or vaccines.

We also endeavored to remain conservative about the clinical and economic benefits of medications or vaccines that reduce the duration of the infectious period. As such we did not account for a direct impact on the health outcomes of those receiving treatment. Such medications or vaccines may potentially reduce the morbidity and mortality associated with COVID-19. Including such benefits would further increase the medication or vaccine’s economic value.

It is important to keep in mind the potential risks and challenges with administering medication or vaccines on a broad scale. For example, typically, medication is prescribed to a focused segment of the population when a doctor can weigh the risks and benefits for individual patients. However, when administering medication more broadly to a diverse set of individuals with varying risk profiles, there may be significant change in the probability of side effects. Additionally, repurposing a medication for SARS-CoV-2 may have unintended consequences, such as limiting its availability for other indications.

## Limitations

All models, by definition, are simplifications of real life and cannot account for every event or outcome. Our model inputs drew from various sources, and new data on SARS-CoV-2 continues to emerge. Sensitivity analyses helped determine the impact of uncertainty and variability in the available data. While our model assumes the infectiousness changes over the duration of the infectious period, infected persons may reduce their number of effective contacts (e.g., isolation) after showing symptoms or progressing to more severe disease. This means that very small reductions at the end of the infectious period may have diminished effects. Nonetheless, the more the infectious period is reduced, the more the periods of high infectiousness will be affected. Our model includes the cost of a treatment course but does not specifically include costs associated with achieving treatment coverage (e.g., outreach campaigns and contact tracing programs). There may be additional costs to have patients tested early enough in order for them to reap all of the benefits of a medication or vaccine that could reduce the duration of the infectious period by 3.5 days. Well-characterized tests that are competitively priced are necessary for a treatment strategy to be successful and economically viable (i.e. not over or under treating the population).

## Conclusion

Our study suggests that finding ways to reduce the infectious period of SARS-CoV-2 could help decrease its spread and impact.

## Methods

### Model structure

Using Microsoft Excel (Microsoft Corporation, Redmond, WA) with the Crystal Ball add-in (Oracle Corporation, Redwood Shore, CA), we developed a computational model representing the U.S. population (327,167,434 persons) and their different interactions with each other as well as the spread of SARS-CoV-2 and the potential health and economic outcomes (S1 Model). The model consists of a stochastic compartment model (i.e., each parameter draws from a distribution) that represent the U.S. population and their mixing patterns and transmission of the virus. Each person who gets infected in this transmission model then travels through a probability tree to simulate what may happen to the person after getting infected, including the various possible health outcomes and resource use. The model advances in discrete, one-day time steps over the course of a year. On any given day, each member of the U.S. population could be in one of five mutually exclusive SARS-CoV-2 compartments: 1) susceptible (S, not infected and able to become infected), 2) exposed (E, infected, but not able to transmit to others), 3) infectious and asymptomatic (I_a_, infected, but without symptoms, and able to transmit to others), 4) infectious and symptomatic (I_s_, infected, showing symptoms, and able to transmit to others), or 5) recovered/immune (R, not infected and unable to become infected). On day one, a set number of individuals start in the ‘I_a_ compartment’ and ‘I_s_ compartment’ to start (i.e., seed) coronavirus, with the remainder starting in the ‘S compartment’. The model assumes equal mixing across all ages and geographic locations in the population each day, and that those who are infectious can potentially transmit the virus to people who are susceptible. Susceptible people can move from the ‘S compartment’ to the ‘E compartment’ if they come in contact with those in the infectious compartment. The following equation determines the number of susceptible individuals who became exposed each day: β*S*I_s_ + (β*0.5)*S*I_a_. Beta (β) equals the basic reproduction number (R_0_; the average number of secondary cases generated by one infectious case) divided by the infectious period duration and the number of individuals in the population, ‘S’ and ‘I’ represent the number of susceptible and infectious persons, respectively, on any given day. We assumed asymptomatic individuals were half as infectious as symptomatic individual, based on available literature.[6, 7] Exposed individuals remain in the ‘E compartment’ for the latent period duration (i.e., time between exposure and ability to transmit) before becoming infectious and moving to the ‘I compartment’ (at a rate of 1/latent period duration). As individuals can transmit the virus prior to disease onset[8], we assume they could transmit one day prior to the start of symptoms. Each simulation the model draws an infectious period duration from a distribution (range: 4–15 days, including day prior to symptom onset, for an average infectious period duration of 9.5 days). Studies show that the viral load of patients began increasing before symptoms began[9], peaked at symptom onset [10, 11] and then quickly declined within 14 days after symptom onset. [9, 12] Therefore, our representation of an infected person's infectiousness over time attempted to replicate this curve with 44% of the transmission occurring by the peak, 43% occurring between the infectiousness peak and halfway to the end of the infectious period and transmission further decreasing for the remainder of the infectious period duration. Infectious individuals remain in the ‘I compartment’ until they recover and are no longer infectious, moving from the ‘I compartment’ to the ‘R compartment’ (at the rate of 1/infectious period duration).

Each symptomatically infected person (i.e., COVID-19 case with an age based on the age distribution of U.S. cases[13]) travels through a probability tree of different sequential outcomes. An infected person showing symptoms starts with a mild infection and has a probability of seeking ambulatory care or calling his/her physician (i.e., telephone consult). This person then has a probability of progressing to severe disease requiring hospitalization. If this person has only mild illness and is not hospitalized, he/she self-treats with over-the-counter (OTC) medications and misses school or work for the duration of symptoms. If the person is hospitalized, he/she has a probability of developing severe pneumonia or severe non-pneumonia symptoms. Also, if the person is hospitalized, he/she also has a probability of intensive care unit (ICU) admission. This patient then has a probability of having either sepsis or acute respiratory distress syndrome (ARDS), with or without sepsis. If this patient has ARDS, he/she requires the use of a ventilator. If the person is hospitalized, he/she has a probability of dying from coronavirus complications. The person accrues relevant costs and health effects as he/she travels through the model. For example, for only mild illness, costs include either ambulatory care or a telephone consult and OTC medications. For hospitalized cases, either ambulatory care or a telephone consult and hospitalization are included. The case incurs the cost of hospitalization associated with the highest ward level of care received (e.g., if admitted to the ICU, incurs the cost of only the ICU-related diagnosis–either sepsis or ARDS–but not the general ward stay) as well as the most severe clinical outcome (e.g., if the patient has ARDS, incurs the cost of ARDS to account for ventilator use, regardless of sepsis).

The third-party payer perspective includes direct costs (e.g., medication, hospitalization), while the societal perspective includes direct and indirect (i.e., productivity losses) costs. Hourly wage across all occupations[14] serves as a proxy for productivity losses. Absenteeism results in productivity losses for the duration of symptoms. Death results in the net present value of productivity losses for missed lifetime earnings based on annual wage[14] multiplied by years of life lost based on an individual’s life expectancy.[15]

### Data sources

Table 1 shows key model input parameters, values, and sources. All costs, clinical probabilities, and durations were age-specific when available and come from scientific literature or nationally representative data sources. Age-specific COVID-19 data are specific to the U.S. context as of March 16, 2020.[13] We report all costs in 2020 values, using a 3% discount rate. We parameterized seeding symptomatic and asymptomatic SARS-CoV-2-infected persons into the population for a given R_0_ such that simulated cases reflected case data reported as of March 24, 2020.[13]

**Table 1:** Model input parameters, values, and sources.

Parameter	Mean or Median	Standard Error or Range	Source
**SARS-CoV-2 Transmission**			
Incubation period (days)	5.2	4.1–7.0	[16]
Infectious period (days)		4–15	[12, 17]
**Costs (2020 US$)**			
Annual wages (all occupations)	40,993	21,950–104,403^a^	[14]
COVID-19 diagnostic test	$51.33		[18]
Ambulatory care visit		110.43–148.33	[18]
Over the counter medications, daily			
0–12 years old^b^	3.87	2.10	[19]
≥13 years old^c^	0.46	0.17	[19]
Hospitalization for pneumonia^d^			
0–17 years old	12,502.30	1,508.04	[20]
18–44 years old	10,627.15	1,045.06	[20]
45–64 years old	13,718.14	1,238.76	[20]
65–84 years old	12,264.39	478.40	[20]
≥85 years old	10,982.73	518.29	[20]
Hospitalization for severe non-pneumonia (all ages)^e^	6,886.53	1,182.99	[20]
Hospitalization for sepsis^f^			
0–17 years old^g^	22,694.30	1,861.33	[20]
18–44 years old	43,778.39	5,382.40	[20]
45–64 years old	38,734.24	2,725.10	[20]
65–84 years old	30,308.29	1,367.91	[20]
≥85 years old	22,694.30	1,861.33	[20]
Hospitalization for ARDS^h^			
0–17 years old	42,350.58	4,198.97	[20]
18–44 years old	26,210.96	1,558.61	[20]
45–64 years old	19,863.98	453.92	[20]
65–84 years old	18,718.55	335.69	[20]
≥85 years old	16,559.75	754.12	[20]
**Probabilities**			
Asymptomatic infection	0.179	0.155–0.202	[21]
Missing work/school	1.0		Assumption
Ambulatory care			
0–4 years old	0.455	0.098	[22]
5–17 years old	0.318	0.061	[22]
18–64 years old	0.313	0.014	[22]
≥65 years old	0.62	0.027	[22]
Probability of hospitalization, given infection			
0–19 years old	0.016		[13]
20–44 years old	0.143		[13]
45–64 years old	0.208		[13]
65–84 years old	0.292		[13]
≥85 years old	0.313		[13]
Probability of ICU admission, given hospitalization			
0–19 years old	0.0		[13]
20–44 years old	0.1399		[13]
45–64 years old	0.2422		[13]
65–84 years old	0.3048		[13]
≥85 years old	0.2013		[13]
Probability of mortality			
0–19 years old	0.0		[13]
20–44 years old	0.007		[13]
45–64 years old	0.0456		[13]
65–84 years old	0.1109		[13]
≥85 years old	0.3323		[13]
Pneumonia, given hospitalization	0.79	0.711-.869^i^	[23]
ARDS, requiring ventilator use in ICU	0.73	0.1697	[24, 25]
Age-group, given infection			
0–19 years old	0.0502		[13]
20–44 years old	0.2879		[13]
45–64 years old	0.3503		[13]
65–84 years old	0.2528		[13]
≥85 years old	0.0588		[13]
**Durations (days)**			
Ambulatory care	0.5		Assumption
Telephone consult (tele-med; minutes)	17	10–24^j^	[26]
Duration of symptoms with mild illness	7	3–17	[27–29]
Duration of symptoms prior to hospital admission	7	3–9^j^	[24, 30]
Hospitalization for pneumonia^d^			
0–17 years old	4.7	0.4	[20]
18–44 years old	4.3	0.4	[20]
45–64 years old	5.1	0.2	[20]
65–84 years old	5.5	0.2	[20]
≥85 years old	5.0	0.2	[20]
Hospitalization for severe non-pneumonia (all ages)^e^	3.1	0.5	[20]
Hospitalization for sepsis^f^			
0–17 years old^g^	7.3	0.5	[20]
18–44 years old	11.2	1.3	[20]
45–64 years old	10.7	0.5	[20]
65–84 years old	8.8	0.4	[20]
≥85 years old	7.3	0.5	[20]
Hospitalization for ARDS^h^			
0–17 years old	9.5	0.75	[20]
18–44 years old	8.8	0.5	[20]
45–64 years old	7.1	0.1	[20]
65–84 years old	7.0	0.1	[20]
≥85 years old	6.1	0.3	[20]
**Utility weights**			
Healthy QALY			
<17 years old	1	-	[31]
18–64 years old	0.92	-	[31]
≥65 years old	0.84	-	[31]
Mild non-specific symptoms^k^	0.648	0.103	[32–41]
Hospitalized, non-pneumonia symptoms^l^	0.514	0.089	[34, 41, 42]
Pneumonia	0.496	0.17	[4, 43–46]
Sepsis	0.467	0.18	[4, 47–52]
ARDS	0.10	0.08–0.15	[4]
**Population**			
U.S. population^m^	327,167,434		[53]

^a^Values are 95% confidence interval

^b^Assumes 5 to 10 mg/kg orally every 6 to 8 hours as needed OR 10 to 15 mg/kg orally every 4 to 6 hours as needed

^c^Assumes 200 mg orally every 4 to 6 hours as needed

^d^Uses International Classification of Diseases, Tenth Revision, Clinical Modification (ICD10) code #J13 Pneumonia due to Streptococcus pneumoniae

^e^Uses International Classification of Diseases, Tenth Revision, Clinical Modification (ICD10) code #J11.89 Influenza due to unidentified influenza virus with other manifestations

fUses International Classification of Diseases, Tenth Revision, Clinical Modification (ICD10) code #R65.21 Severe sepsis with septic shock

^g^Data for age-group unavailable and uses lowest values of all age-groups as a proxy

^h^Uses International Classification of Diseases, Tenth Revision, Clinical Modification (ICD10) code #J96.22 Acute and chronic respiratory failure with hypercapnia for 18 years and older and ICD10 code #J96.20 Acute and chronic respiratory failure, unspecified whether with hypoxia or hypercapnia for 0 to 17-year olds

^i^Values are interquartile range

^j^Values are 10%-90%

^k^Uses influenza without hospitalization as a proxy

^l^Uses influenza with hospitalization as a proxy

^m^2018 population estimate

### Experimental scenarios

The overall goal of this study was to evaluate the potential impact of reducing the infectious period duration from the third-party payer and societal perspectives across a range of possible scenarios, rather than predict exactly what will happen with the current pandemic. Since currently available data may not capture the full extent to which the virus has actually spread and the degree to which different social distancing measures have actually been applied, our team ran ranges of possible epidemic scenarios that varied R_0_ and the proportion of the population that has already exposed to SARS-CoV-2 prior to implementation of the treatment. This could then offer a sense on how the treatment could then affect the transmission under a wide range of circumstances after social distancing measures have been relaxed completely. Experiments consisted of 1,000 trial Monte Carlo simulations, varying parameters throughout their range (Table 1). Our initial scenario assumed all infected individuals would transmit the virus for an average of 9.5 days, including transmission prior to symptom onset. Experimental scenarios consisted of using medication or vaccination to reduce the infectious period duration, thereby moving individuals to the ‘R compartment’ at a faster rate by drawing an infectious period duration from a narrower distribution, decreased by an average of 0.5, 2, and 3.5 days (which results in an average infectious period duration of 9, 7.5, and 6 days, respectively) among those that receive the treatment (i.e., medication or vaccination). The model assumes that patients are treated early enough for reductions in infectiousness to be fully realized in each scenario. The first set of experimental scenarios treated only symptomatic individuals, while the second set treated all infected with SARS-CoV-2. Sensitivity analyses varied the proportion of the population receiving medication or vaccination, ranging from 25% to 75%, in order to show a more aggressive upper bound of potential coverage, when treatment started during the epidemic (after 5–15% of the population has been exposed), total treatment cost ($500-$2,500), and R_0_ (2.0–3.5).[16, 54, 55] Total treatment cost includes treatment research and development, treatment manufacturing (e.g., scaling manufacturing capacity), and costs associated with treatment delivery and administration. Results reported in the text refer to treatment starting when 15% of the population has been exposed, unless otherwise noted.

For each scenario, we calculated its net cost savings:

Net Cost Savings = Direct Cost and Productivity Losses of Averted Infections (Benefit)—Cost of Reducing the Infectious Period Duration (Cost)

ICER = (Cost_With Treatment_-Cost_Without Treatment_)/(Health Effects_Without Treatment_-Health Effects_With Treatment_)

where health effects were measured in quality-adjusted life years (QALYs) lost (i.e., accounting for reductions in health effects due to COVID-19 and/or death). Each infection accrues QALY values based on age-dependent healthy QALY values attenuated by infection outcome-specific utility weights for their infection duration. Healthy QALYs listed in Table 1 assume individuals have no other conditions and are based on a widely accepted national study that derived standard healthy QALYS for all ages.[31] Death results in the loss of the net present value of QALYs for the remainder of his/her lifetime.

## Supporting information

S1 Model Computational model developed by PHICOR team at CUNY Graduate School of Public Health & Health Policy. (XLSX)
